# Inflammatory Leukocyte Phenotypes Correlate with Disease Progression in Idiopathic Pulmonary Fibrosis

**DOI:** 10.3389/fmed.2014.00056

**Published:** 2014-12-22

**Authors:** Bethany B. Moore, Chris Fry, Yueren Zhou, Susan Murray, MeiLan K. Han, Fernando J. Martinez, Kevin R. Flaherty, The COMET Investigators

**Affiliations:** ^1^Department of Internal Medicine, Division of Pulmonary and Critical Care Medicine, University of Michigan, Ann Arbor, MI, USA; ^2^Department of Microbiology and Immunology, University of Michigan, Ann Arbor, MI, USA; ^3^Department of Biostatistics, School of Public Health, University of Michigan, Ann Arbor, MI, USA; ^4^Department of Internal Medicine, Weill Cornell Medical School, New York, NY, USA

**Keywords:** lung, lymphocyte, monocyte, interstitial lung disease, peripheral blood

## Abstract

Idiopathic pulmonary fibrosis (IPF) is characterized by progressive deposition of extracellular matrix, worsening dyspnea, and eventual mortality. Pathogenesis of IPF is poorly understood and the role inflammation and activated leukocytes play in the disease process is controversial. Previous studies demonstrated that activated leukocyte subsets characterize IPF patients. We sought to validate this observation in a well-defined cohort of 35 IPF patients and to correlate the observed leukocyte phenotypes with robust parameters of disease progression. We demonstrate that in univariate and multivariate analyses, increases in the CD14hi, CD16hi subset of monocytes measured at baseline correlated with disease progression, with a threshold value >0.5% of the total peripheral blood mononuclear cells being a significant predictor for worse outcome. In addition, several T cell subsets, including CD25 expressing CD4 cells, and CXCR3 expressing CD4 and CD8 subsets correlated with disease progression when found in increased percentages in the peripheral blood of IPF patients when sampled at baseline. Somewhat surprising in comparison to previous literature, the CD4 T cells did not appear to have lost expression of the co-stimulatory molecule, CD28, but the CD8 T cells did. Taken together, these results are consistent with the presence of an inflammatory process in IPF patients who eventually progress. However, when longitudinal measurements of these same markers were examined, there was significant heterogeneity of expression and these biomarkers did not necessarily remain elevated in IPF patients with progressive disease. We interpret this heterogeneity to suggest that IPF patients experience episodic inflammatory events that once triggered, may lead to disease progression. This longitudinal heterogeneity in biomarker analyses may explain why such markers are not consistently measured in all IPF cohorts.

## Introduction

Idiopathic pulmonary fibrosis (IPF) is a devastating disease of unknown etiology. It is characterized by progressive deposition of extracellular matrix proteins, dyspnea, and eventual mortality. The natural history of the disease is variable, with some patients experiencing relative stability over time, and others experiencing a more rapid decline in lung function. There is an on-going debate about the pathogenesis of IPF. Many investigators believe that the disease results from aberrant epithelial–mesenchymal cell interactions (1, 2). However, there have also been numerous studies suggesting a role for occult viral infections as co-factors for disease development or progression (3). Furthermore, results of immunosuppressive therapies have been mixed in patients with interstitial lung disease. For instance, two studies have reported benefit (4, 5) while a more recent clinical trial demonstrated that immunosuppressive therapy was harmful to these patients (6). This last trial raised the possibility that at least some immune functions may be protective in IPF.

Several past studies have sought to identify a phenotype of circulating leukocytes that correlate with disease progression in IPF. Most notably, previous studies have suggested CD4 T cells in IPF patients display reduced levels of CD28, suggesting persistent antigen activation and perhaps clonal exhaustion of these T helper cells (7). Other studies have shown increased levels of MHC class II and CD40 ligand (CD154) on CD4 cells in IPF patients (8) and these same studies showed evidence of T cell receptor Vβ oligoclonal expansion, again suggesting antigen-specific expansion. These data are further supported by recent gene array analysis of peripheral blood mononuclear cells in IPF and control patients. These genetic studies demonstrated that IPF patients were characterized by a gene expression profile consistent with T cell activation, including enhanced expression of CD28, inducible T cell co-stimulator (ICOS), lymphocyte-specific protein tyrosine kinase (LCK), and IL-2-inducible kinase (ITK), which are all T cell co-stimulatory molecules (9). Past studies have also looked at CD25 expressing T cells. CD25 is the IL-2 receptor and this protein is upregulated on both activated T cells and on T regulatory (Treg) cells. While two studies have found elevated levels of Tregs in IPF patients (10, 11), two other studies have had opposite results (12, 13). However, in the study by Kotsianidis et al., the function of the Tregs in IPF patients was found to be impaired. Taken together, these data suggest that IPF patients are characterized by pro-inflammatory and activated T cell phenotypes.

The ability of activated T cells to migrate into tissues is controlled by chemokine receptors. Additionally, the chemokine receptor profile correlates with the nature of the T cell activation. The expression of CXCR3 on lymphocytes is believed to correlate with Th1 responses while the expression of CCR4 is believed to correlate with Th2 responses (14). A previous study looked at the ratio of CXCR3 to CCR4 expressing lymphocytes in the lung tissue of patients diagnosed with IPF vs. non-specific interstitial pneumonitis (NSIP) and found that NSIP patients, which have a better prognosis than IPF patients had a ratio of CXCR3 > CCR4, whereas IPF patients had a ratio that was approximately even (15). These data suggested that the T cell activation in IPF patients is likely skewed toward a Th2 response. Not surprisingly, the Th2 cytokines IL-4 and IL-13 are well known to promote fibrogenesis in both animal models and human studies (16, 17).

Finally, there has been growing interest in phenotypes of circulating monocytes in homeostasis and disease. In the mouse, a population of Ly6Chi, Gr-1+ monocytes have been characterized, which express high levels of CCR2, but low levels of CX3CR1 (the fractalkine receptor). These cells are believed to be inflammatory monocytes with increased phagocytic capacity, lower cytokine expression, and a tendency to make IL-10 in response to lipopolysaccharide (LPS) stimulation (18, 19). Ly6Chi monocytes can facilitate alternative activation of macrophages during fibrogenesis and depletion of this subset in mice can limit lung collagen deposition (20). In human beings, this population is characterized as CD14hi, CD16lo. In contrast, the murine population, which is characterized as Ly6Clo, Gr-1− is characterized by high expression of CX3CR1, low CCR2 and this population of cells is considered pro-inflammatory because they secrete TNFα and IL-1 in response to bacterial LPS (18, 19, 21). This population can also promote fibrosis as it has been shown to express vascular endothelial growth factor, to promote myofibroblast differentiation, and to increase collagen deposition (22). This population is CD14hi CD16hi in human beings. Recent studies have shed light on the role that various monocyte populations may play in the pathogenesis of lung fibrosis by showing that circulating monocytes can be a source of profibrotic matricellular proteins (23, 24) and alternative activation of monocytes has been linked previously to IPF (20, 23) and experimental lung fibrosis (25, 26).

The “correlating outcomes with biochemical markers to estimate time-progression in IPF” (COMET) study was a longitudinal observational study undertaken to assess whether readily accessible biomarkers could be identified that correlated with disease progression. In this study, a cohort of 35 IPF patients had peripheral blood collected for immunophenotyping analysis. We chose to examine T cell and monocyte compartments based on the literature described above to determine whether we could validate any of these measures as markers of disease progression. Overall, our results suggest that activated T cell and monocyte phenotypes characterize progressive IPF.

## Materials and Methods

### Patient enrollment

The COMET study was a multi-center, observational cohort study of well-defined IPF patients followed prospectively at 16-week intervals up to 80 weeks (http://www.clinicaltrials.gov, clinical trial ID no. NCT01071707). Patients were diagnosed as having IPF on the basis of characteristic CT scans or UIP pathology confirmed by lung biopsy. All subjects underwent baseline assessment, including demographics, patient-reported descriptors, spirometry, diffusing capacity of the lung for CO (DLco), 6-min walk testing (6MWT), and high-resolution computed tomography. Patients were allowed to remain on current treatments. The primary outcome (combined endpoint) was progression-free (PF) survival as determined by the time until any of the following: death, acute exacerbation of IPF, lung transplant, or relative change in forced vital capacity (FVC, liters) of ≥10% or DL_CO_ (ml min^−1^ mmHg^−1^) of 15%. Each site received local Institutional Review Board approval. Two previous studies have reported on data collected from the COMET cohort (24, 27).

### Sample preparation and flow cytometry characterization

Peripheral blood was collected in EDTA-containing vacutainers at study centers and samples were shipped by overnight mail using cold packs to the University of Michigan. Whole blood was centrifuged at 2500 rpm for 10 min and plasma was collected. Blood cells were diluted in sterile saline and subjected to centrifugation on Ficoll-hypaque to obtain the PBMC fraction. Cells were washed, counted, and stained for flow cytometry using the following parameters. Cells were stained at 1.0 × 10^6^/well with either parameter antibodies or IgG controls.

#### Parameter 1

APC Mouse anti-Human CD4 (BD Biosciences), FITC Mouse Anti-Human CD8 (BD), PE Mouse Anti-Human CD28 (BD), V450 Mouse Anti-Human CD194 (CCR4) (BD), Alexa Fluor 700 Mouse anti-Human CD183 (CXCR3) (BD), and PerCP-Cy5.5 Mouse anti-Human CD19 (BD).

#### Parameter 2

APC Mouse anti-Human CD4 (BD), FITC mouse anti-Human HLA-DR (class II) (BD), PE mouse Anti-Human CD154 (BD), and PerCp-Cy 5.5 mouse Anti-Human CD25 (BD).

#### Parameter 3

PE-Cy7 Mouse anti-Human CD14 (BD), PE Mouse Anti-Human CD16 (BD), APC Rat anti-human TLR-9 (BD), FITC Mouse Anti-Human CD64 (BD), APC Cy7 anti-Human CD206 (Biolegend, San Diego, CA, USA), and PerCP-Cy5.5 anti-Human CD192 (CCR2) (Biolegend).

#### Parameter 4

PE-Cy7 Mouse anti-Human CD14 (BD), APC Cy7 anti-Human CD206 (Biolegend, San Diego, CA, USA). Following staining cells underwent further intra-cellular staining as described by BD biosciences cytofix/cytoperm intra-cellular staining kit for APC Rat anti-human TLR-9 (BD), and FITC anti-Human Fractalkine/CX3CL1 (R&D systems, Minneapolis, MN, USA).

##### IgG controls

PE mouse IgG1(BD), APC Rat IgG2a (BD), PerCp/Cy5.5 mouse IgG2b (Biolegend), Alexa Fluor 700 Mouse IgG1 (BD), PE-Cy7 Mouse IgG2a (BD), V450 Mouse IgG1 (BD), APC Cy7 Mouse IgG1 (Biolegend), FITC Mouse IgG2a (BD).

Following staining, cells were fixed with 2% paraformaldehyde and run on a BD Biosciences LSR II flow cytometer (San Jose, CA, USA). Data were analyzed using Winlist 6.0 (Topsham, ME, USA). Results are reported as the percent of the phenotype being analyzed in the total peripheral blood mononuclear cell pool.

### Statistical analyses

Demographics, pulmonary function, and biomarker levels are compared between known progressors and non-progressors over 80 weeks of follow-up using Wilcoxon rank-sum tests for continuous predictors and Fisher’s exact test for dichotomous predictors. Additionally, boxplots were generated to provide a visual comparison between COMET progressors and non-progressors for some biomarker levels tested. Four patients of the 35 patients enrolled in this COMET study were excluded from this descriptive analysis when classification by progressor status was unclear based on incomplete follow-up data. However, no exclusions of this nature were made when conducting time-to-event analyses described below. Univariate Cox proportional hazards models were fit to examine the association between each biomarker level and reaching the combined endpoint. In order to compare impact of biomarker levels in the various models, standardized biomarker levels were calculated by subtracting mean levels and subsequently dividing by the biomarker level SD. The standardization allows us to interpret each hazard ratio (HR) in terms of a 1 SD increase in the biomarker level. In addition, optimized threshold values for each biomarker were determined by the function cox.main from the R AIM package and then subsequently assessed for association with the combined endpoint. These analyses were repeated adjusting for baseline characteristics including age, gender, FVC%, DL_CO_%, desaturation <88% during 6MWT, history of gastroesophageal reflux disease and smoking status. The index of concordance is a measure of model fit based on the probability of correctly predicting which patient will achieve the combined endpoint first when considering a pair of patients from the dataset. This index was used to determine whether the original biomarker level or the threshold representation achieved better model fit. Longitudinal trajectories of biomarker levels over time are displayed by progressor vs. non-progressor status with a red dashed line indicating thresholds identified previously using the R AIM package as described above. Kaplan–Meier curves depict the PF survival probability (combined endpoint) between subjects whose biomarker level is above the identified threshold and those below in a small time interval. The Kaplan–Meier curve assumes that censoring is independent and can only occur at the right of the time interval. All analyses are conducted in an exploratory manner and therefore statistical significance as measured by *p*-values must be taken in that context. That is, for every 20 statistical *p*-values displayed in this manuscript, we would expect 1 comparison on average to spuriously give a *p*-value of <0.05 due to the statistical nature of exploratory analyses with multiple comparisons. Significant *p*-values are indicated by asterisk in all tables.

## Results

### Baseline clinical characteristics of progressors vs. non-progressors

Of the 35 COMET patients analyzed in these studies, 22 met criteria for progression and 9 remained stable. Four patients were excluded from these analyses due to incomplete follow-up. Table 1 shows the baseline characteristics of COMET participants by disease progression status. This table also presents the mean values for various leukocyte phenotypes arranged according to monocyte, CD4 and CD8 phenotypes. In addition, levels of CXCR3 and CCR4 staining on T cells are presented. There were no significant differences in the age, DL_CO_, FVC, or FEV1 measurements made at baseline between the two groups. As expected, there was a slight male predominance in both groups. Although the progressive patients tended to have a higher history of smoking and gastro esophageal reflux disease, these features did not reach significance. The functional parameter that was significantly different between these two groups was the tendency to desaturate during a 6MWT. 54.5% of the progressive patients desaturated during this procedure whereas only 11.1% of the stable patients did so.

**Table 1:** **Demographics, pulmonary function, and biomarker levels for patients stratified on the basis of disease progression**.

	Non-progressors				Progressors				
Parameters of interest	*n*	Mean	SD	Range	*n*	Mean	SD	Range	*p*-Value
Age	9	65.7	7.9	53.0–75.0	22	64.8	8.9	44.0–78.0	0.84
DLCO	9	45.8	14.6	16.4–64.7	22	42.9	12.3	22.6–69.7	0.27
FVC	9	72.2	13.9	51.4–83.8	22	70.9	15.0	42.4–97.9	0.81
FEV1	9	77.2	19.3	53.4–98.1	22	73.9	15.9	44.1–107.4	0.84
Male	9	5 (55.6%)			22	13 (59.1%)			0.99
Past smoker	9	4 (44.4%)			22	14 (63.6%)			0.43
Desaturation	9	1 (11.1%)			22	12 (54.5%)			0.045*
History of gastroesophageal reflux disease	9	4 (44.4%)			22	13 (59.1%)			0.69
**MONOCYTE PHENOTYPING**									
**CD14**	3	3.5	0.7	2.7–3.9	10	19.3	21.2	1.2–65.6	0.15
CD14 CD206	5	1.3	0.8	0.3–2.5	14	2.7	2.5	0.5–9.9	0.29
CD14 TLR9	5	4.9	7.5	0.3–17.8	5	2.5	1.5	0.1–4.2	0.99
CD14 fractalkine	5	0.5	0.3	0.1–0.8	5	2.1	3.3	0.1–7.9	0.40
**C14hi CD16lo**.	9	2.8	1.5	0.1–5.0	22	3.5	2.2	0.7–7.6	0.65
CD14hi CD16lo CD64	9	2.7	1.5	0.02–4.4	22	3.4	2.2	0.2–7.6	0.68
CD14h CD16lo CCR2	9	2.2	1.7	0.01–4.8	22	3.3	2.1	0.4–7.3	0.27
**CD14hi CD16hi**	7	1.8	3.7	0.07–10.2	22	2.5	8.2	0.03–39.3	0.52
CD14hi CD16hi CD64	7	1.7	3.7	0.05–10.1	22	2.4	8.2	0.03–39.3	0.59
CD14hi CD16hi CCR2	7	1.7	3.7	0.002–10.2	22	2.4	8.3	0.03–39.3	0.63
**CD4 PHENOTYPING**									
**CD4**	9	6.9	3.0	3.8–12.4	22	8.4	3.9	1.0–18.1	0.29
CD4 CD28hi	9	5.3	1.8	3.4–8.7	22	6.0	4.5	0.1–17.6	0.62
CD4 CD28lo	9	1.5	2.1	0.06–5.5	22	2.4	3.6	0.04–13.8	0.59
CD4 CD25	9	0.3	0.2	0.09–0.7	22	1.55	1.6	0.01–6.6	0.052
CD4 MHC class II	9	0.5	0.4	0.01–0.49	22	1.65	2.3	0.01–3.94	0.14
CD4 CD154	9	0.19	0.16	0.01–0.5	22	0.65	0.89	0.01–3.95	0.23
**CD8 PHENOTYPING**									
**CD8**	9	4.5	6.9	0.5–22.1	22	3.3	2.0	1.1–8.1	0.33
CD8 CD28hi	9	1.3	1.4	0.3–4.6	22	1.0	0.8	0.1–2.6	0.98
CD8 CD28lo	9	3.2	5.6	0.2–17.5	22	2.3	1.9	0.3–6.8	0.31
**CCR4 AND CXCR3 ANALYSES ON T CELLS**									
**CCR4**	5	7.4	12.3	0.06–28.9	18	7.0	5.8	0.2–19.8	0.33
CCR4 CD4	4	3.3	3.9	0.1–8.6	18	2.6	2.7	0.1–8.6	0.97
CCR4 CD8	5	0.5	0.7	0.01–1.8	18	1.1	0.8	0.01–2.8	0.17
**CXCR3**	5	5.9	6.6	0.9–16.6	18	14.1	12.4	1.2–46.2	0.11
CXCR3 CD4	5	2.1	2.1	0.3–4.6	18	5.7	4.2	0.05–12.3	0.15
CXCR3 CD8	5	0.5	0.4	0.2–1.2	18	2.9	6.0	0.1–26.5	0.03*

### Progressive IPF patients have higher percentages of CD4 CD25 and CD8 CXCR3 expressing cells

**Figure 1:**
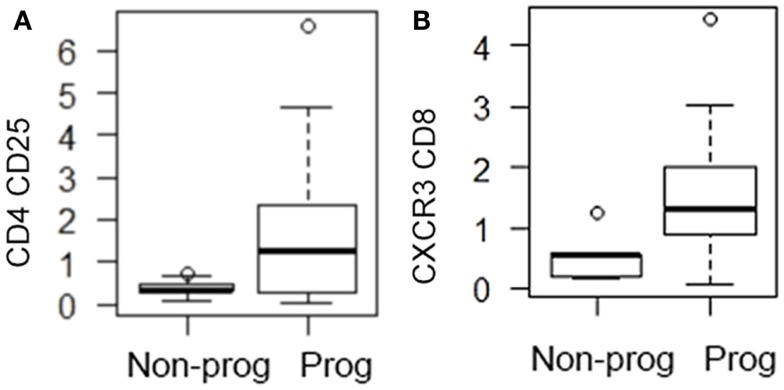
**Progressive IPF patients are characterized by higher percentages of CD4 CD25+ and CD8 CXCR3+ cells**. The box shows the range of the data from 25th percentile to 75th percentile. The bar in the middle of the box represents the median (50th percentile). The lower and upper whiskers gives the range of the data with each whisker restricted to 1.5 times the interquartile range (which is defined as the upper quartile minus the lower quartile) with circles indicating data outside of this whisker range. **(A)** CD4 CD25 percentages are graphed; **(B)** CXCR3 CD8 percentages are graphed.

**Figure 2:**
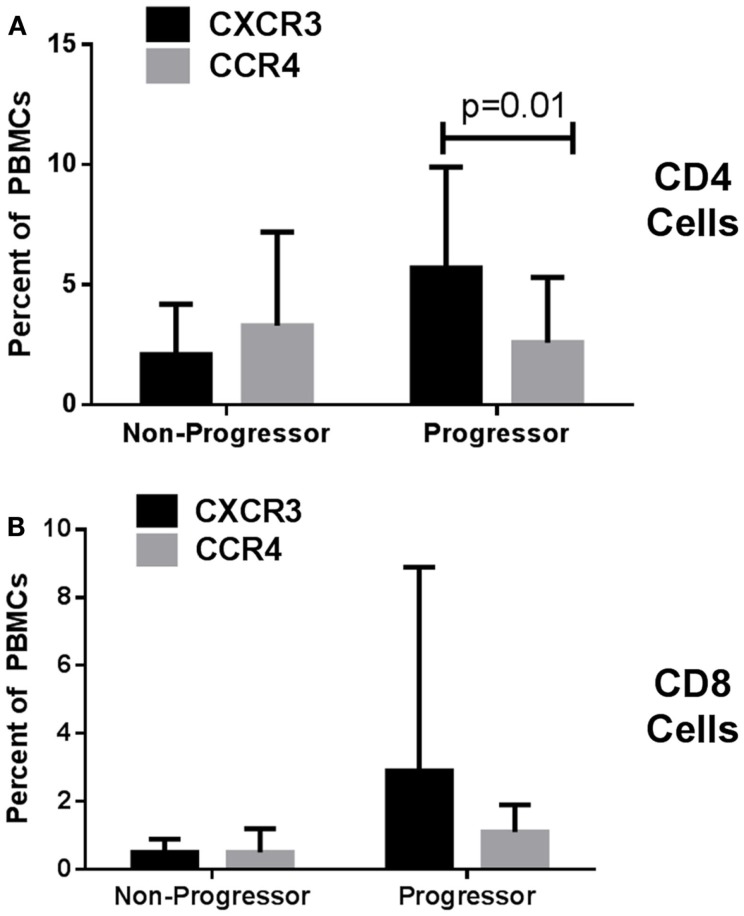
**Idiopathic pulmonary fibrosis patients have higher percentages of CXCR3 than CCR4 expressing CD4 T cells**. Mean values of each cell type were analyzed by unpaired *T* test adjusting for multiple comparisons using the Holm–Sidak method. **(A)** graphs CD4 cells while **(B)** graphs CD8 cells.

Looking at the baseline biomarker characteristics, only two analyses were significantly different or approached significance (*p* = 0.052) between progressors and non-progressors. There were more CD4 CD25 and CXCR3 CD8 expressing T cells in the progressive cohort when compared to the stable IPF patients (Table 1). Figure 1 shows boxplots graphically representing the percentages of these cell types identified at baseline in the stable vs. progressive IPF patients. On average, the progressive patients had fivefold more CD4 CD25 cells. Similarly, the progressive patients had ~5.8-fold higher percentages of CXCR3 expressing CD8 cells. The progressive IPF patients have mean values of CXCR3 expressing CD4 and CD8 T cells that are much greater (~2-fold or more) than their mean values of CCR4 expressing cells (Table 1). This difference reached significance for the CD4 subset (Figure 2). In contrast, the ratios are more equivalent in the stable IPF patients.

### Inflammatory T cell and activated monocytes predict IPF progression in univariate analyses

The univariate associations between the percentages of each biomarker in the flow cytometry analyses of the peripheral blood leukocytes and the likelihood of reaching the combined endpoint for disease progression are shown in Table 2. A 1 SD increase in the percentage of monocytes expressing high levels of both CD14 and CD16 were found be to significantly associated with reaching the combined endpoint with a hazard ratio (HR) 2.13 (95% CI 1.10–4.15), *p* = 0.026. In our analyses, essentially all of these monocytes expressed both CD64, an Fc receptor upregulated during cellular activation and CCR2, an inflammatory chemokine receptor. Interestingly, the classic inflammatory macrophage subset (CD14hi, CD16lo, CCR2+) was also associated with disease progression. Patients with a 1 SD increase in the percentage of this subset had a HR of 1.57 (95% CI 0.99–2.51) for meeting the combined endpoint for disease progression and this result approached significance (*p* = 0.056).

**Table 2:** **Univariate Cox model results based on standardized biomarker level values**.

Parameters of interest	*n*	HR	95% CI	*p*-Value	Index of concordance
**MONOCYTE PHENOTYPES**					
CD14	13	1.32	0.80–2.17	0.278	0.720
CD14 CD206	22	1.29	0.87–1.93	0.208	0.602
CD14 TLR9	12	0.76	0.24–2.34	0.627	0.395
CD14 fractalkine	12	1.91	0.73–4.99	0.185	0.605
C14hi CD16lo	35	1.43	0.89–2.29	0.141	0.591
CD14hi CD16lo CD64	35	1.43	0.89–2.32	0.142	0.589
CD14hi CD16lo CCR2	35	1.57	0.99–2.51	0.056	0.638
CD14hi CD16hi	33	2.13	1.10–4.15	0.026*	0.593
CD14hi CD16hi CD64	33	2.20	1.10–4.38	0.025*	0.584
CD14hi CD16hi CCR2	33	2.10	1.09–4.06	0.027*	0.584
**CD4 PHENOTYPES**					
CD4	35	1.43	0.94–2.17	0.099	0.618
CD4 CD28hi	35	1.27	0.82–1.95	0.283	0.598
CD4 CD28lo	35	1.15	0.78–1.70	0.471	0.553
CD4 CD25	35	2.13	1.37–3.33	0.0008*	0.687
CD4 MHC class II	35	1.18	0.87–1.61	0.277	0.581
CD4 CD154	35	1.2	0.89–1.62	0.231	0.581
**CD8 PHENOTYPES**					
CD8	35	1.00	0.71–1.42	0.981	0.654
CD8 CD28hi	35	1.04	0.72–1.52	0.821	0.600
CD8 CD28lo	35	1.00	0.70–1.43	0.982	0.370
**CCR4 AND CXCR3 ANALYSES**					
CCR4	26	0.94	0.57–1.57	0.827	0.529
CCR4 CD4	25	0.93	0.58–1.48	0.753	0.511
CCR4 CD8	26	0.89	0.58–1.37	0.610	0.420
CXCR3	26	2.42	1.47–3.97	0.0005*	0.706
CXCR3 CD4	26	1.73	1.12–2.67	0.014*	0.662
CXCR3 CD8	26	33.72	4.21–270.32	0.001*	0.775

When looking at the percentage of CD4 cells that were noted in the peripheral blood of IPF patients, progressive patients tended to have more of every subtype (Table 1) although as mentioned above, the only subset to approach significance was the CD4 CD25 subset. Univariate analyses showed a strong significance for patients with a 1 SD increase in the number of CD4+CD25+ cells experiencing disease progression, HR 2.13 (95% CI 1.37–3.33), *p* = 0.0008.

### Results of univariate threshold-based-analyses demonstrate that increased levels of monocyte and T cell subsets predict progression

**Figure 3:**
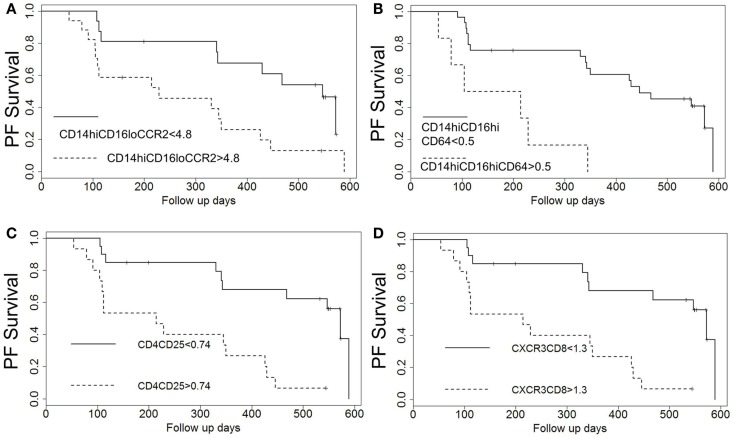
**Kaplan–Meier plots show decreased probability of progression-free (PF) survival for patients with elevated percentages of T cell and monocyte subsets above identified thresholds**. Patients with elevated percentages of **(A)** CD14hi CD16lo CCR2+ monocytes, **(B)** CD14hi, CD16hi, CD64+ monocytes, **(C)** CD4 CD25 T cells, and **(D)** CXCR3 CD8 T cells above the indicated thresholds identified in Table 3 show worse probability of PF survival.

We next wanted to determine whether thresholds for each biomarker could be established that could predict which patients would go on to progress. Results of univariate threshold-based analyses of biomarker levels are shown in Table 3. Interestingly, in this analysis, patients with CD14 monocyte percentages >4 had a HR of 12.41 (95% CI 1.49–103.22), *p* = 0.02. Analyzing which populations of these monocytes had the highest predictive value, both inflammatory CD14hi CD16lo and CD14hi CD16hi subsets were strongly correlated with progressive disease. Figure 3A shows the Kaplan–Meier plots analyzing the probability of PF survival for patients with inflammatory monocytes (CD14hi CD16lo CCR2+) found at thresholds above and below 4.8% of the peripheral blood pool. Similarly, Figure 3B shows the Kaplan–Meier plots analyzing CD14hi, CD16hi, CD64+ monocytes at thresholds below and above 0.5% of the peripheral blood pool. Collectively, these data show that patients with elevated percentages of these markers have poor PF survival as judged by meeting the combined endpoint.

**Table 3:** **Univariate threshold-based analyses of biomarker levels and association between reaching the combined endpoint**.

Parameters of interest	*n*	HR	95% CI	*p*-Value	Index of concordance
**MONOCYTE THRESHOLDS**					
CD14 > 4	13	12.41	1.49–103.22	0.020*	0.740
CD14 CD206 > 1.8	22	2.72	0.93–7.96	0.068	0.620
CD14 TLR9 > 1.3	12	3.87	0.43–34.9	0.228	0.686
CD14 fractalkine > 0.7	12	2.26	0.37–13.8	0.376	0.628
C14hi CD16lo > 5	35	5.54	1.97–15.53	0.001*	0.620
CD14hi CD16lo CD64 > 4.6	35	2.86	1.15–7.12	0.024*	0.623
CD14hi CD16lo CCR2 > 4.8	35	5.54	1.97–15.53	0.001*	0.620
CD14hi CD16hi > 0.5	33	2.45	1.01–5.94	0.047*	0.627
CD14hi CD16hi CD64 > 0.5	33	2.77	1.17–6.55	0.020*	0.648
CD14hi CD16hi CCR2 > 0.2	33	0.85	0.36–2.02	0.717	0.485
**CD4 THRESHOLDS**					
CD4 > 10	35	2.08	0.84–5.12	0.112	0.603
CD4 CD28hi > 8.7	35	3.92	1.55–9.92	0.004*	0.621
CD4 CD28lo > 4.7	35	0.92	0.31–2.73	0.883	0.521
CD4 CD25 > 0.74	35	4.73	1.86–12.05	0.001*	0.678
CD4 MHC class II > 0.11	35	0.65	0.19–2.22	0.494	0.526
CD4 CD154 > 0.03	35	2.65	0.3–19.76	0.342	0.528
**CD8 THRESHOLDS**					
CD8 > 2	35	2.68	0.98–7.29	0.054	0.633
CD8 CD28hi > 1.8	35	2.63	0.95–7.31	0.063	0.592
CD8 CD28lo > 1.9	35	2.43	1.03–5.74	0.043*	0.605
**CCR4 AND CXCR3 THRESHOLDS**					
CCR4 > 6	26	0.66	0.27–1.65	0.376	0.584
CCR4 CD4 > 7.6	25	0.95	0.21–4.19	0.942	0.526
CCR4 CD8 > 2.7	26	0.30	0.04–2.25	0.242	0.546
CXCR3 > 7.5	26	2.58	1.00–6.68	0.050	0.616
CXCR3 CD4 > 10.3	26	2.98	0.96–9.3	0.060	0.578
CXCR3 CD8 > 1.3	26	17.12	3.49–83.96	0.00046*	0.712

When looking at the T cell markers, higher percentages of CD8 cells and CXCR3 expressing T cells were all positively associated with an increased risk of disease progression. In addition, levels of CD4 CD25 cells above the threshold of 0.74% and levels of CD4 CD28hi T cells above 8.7% predicted worse outcomes. Figures 3C,D show the Kaplan–Meier plot showing the decreased probability of PF survival for patients with CD4 CD25 expressing cells below and above 0.74% and for patients with CXCR3 CD8 cells above and below 1.3%, respectively.

### Multivariate analyses confirm inflammatory and activated leukocytes predict poor outcomes in IPF patients

We next performed a series of multivariate analyses: Table 4 shows corresponding results when adjusted for age, gender, FVC%, DL_CO_%, desaturation <88% during 6MWT, history of gastroesophageal reflux disease and smoking status. Table 5 provides threshold analyses that are adjusted for the factors listed above. In multivariate analyses, our results demonstrated continued significance for a change of 1 SD in the percentages of the CD14hi CD16hi monocyte subset correlating with disease progression. However, only the CD14hi, CD16hi, CD64 expressing subset maintained significance in the threshold analyses suggesting that percentages above 0.5% predict poor outcomes. This is also supported by the Kaplan–Meir plots in Figure 3B. Although not significant in the multivariate Cox-models, a threshold above which all of the CD14hi CD16lo subsets could predict disease progression was noticeable in the multivariate analyses. Consistent with the concept that IPF patients have activated lymphocyte profiles, higher levels of CD4 cells (>10% of total peripheral blood) or increases in CD8 cells >2% in the peripheral blood marked patients at risk of functional decline. In the threshold analyses, it appears the T cells maintaining high levels of CD28 have the highest predictive value. The associations with higher CXCR3 expressing CD8 cells correlating with disease progression were maintained in every analysis. This is also consistent with the Kaplan–Meier analyses in Figure 3D.

**Table 4:** **Cox model results based on standardized biomarker level values adjusted for age, gender, dlco10%, fvc10%, desaturation, gastroesophageal reflux disease and smoke status**.

Parameters of interest	*n*	HR	95% CI	*p*-Value	Index of concordance
**MONOCYTE PHENOTYPES**					
CD14	13	6.51E-08	0.0017–44.49	0.219	0.973
CD14 CD206	22	0.95	0.47–1.90	0.875	0.801
CD14 TLR9	12	239.80	1.2e-11-4.8e15	0.726	1.000
CD14 fractalkine	12	0.44	5.5e-18-3.5e16	0.967	1.000
C14hi CD16lo	35	1.36	0.82–2.26	0.232	0.738
CD14hi CD16lo CD64	35	1.42	0.85–2.38	0.177	0.744
CD14hi CD16lo CCR2	35	1.52	0.91–2.56	0.112	0.746
CD14hi CD16hi	33	2.59	1.15–5.86	0.022*	0.730
CD14hi CD16hi CD64	33	2.67	1.17–6.06	0.019*	0.721
CD14hi CD16hi CCR2	33	2.67	1.17–6.11	0.020*	0.723
**CD4 PHENOTYPES**					
CD4	35	1.66	0.99–2.78	0.053	0.764
CD4 CD28hi	35	1.77	1.08–2.88	0.022*	0.770
CD4 CD28lo	35	0.96	0.61–1.53	0.871	0.722
CD4 CD25	35	1.78	1.04–3.03	0.035*	0.758
CD4 MHC class II	35	0.99	0.67–1.47	0.096	0.728
CD4 CD154	35	1.06	0.71–1.57	0.785	0.734
**CD8 PHENOTYPES**					
CD8	35	1.13	0.70–1.84	0.611	0.730
CD8 CD28hi	35	1.17	0.71–1.95	0.537	0.740
CD8 CD28lo	35	1.11	0.68–1.82	0.663	0.724
**CCR4 AND CXCR3 PHENOTYPES**					
CCR4	26	0.64	0.33–1.26	0.201	0.730
CCR4 CD4	25	0.73	0.43–1.24	0.247	0.734
CCR4 CD8	26	0.76	0.40–1.44	0.400	0.734
CXCR3	26	2.14	1.18–3.90	0.012*	0.782
CXCR3 CD4	26	1.78	0.91–3.46	0.090	0.754
CXCR3 CD8	26	125.23	5.00–3133.5	0.003*	0.792

**Table 5:** **Threshold-based analyses of biomarker levels and association between reaching the combined endpoint adjusted for age, gender, dlco10%, fvc10%, desaturation, gastroesophageal reflux disease, and smoke status**.

Parameters of interest	*n*	HR	95% CI	*p*-Value	Index of concordance
**ADJUSTED MONOCYTE THRESHOLDS**					
CD14 > 4	13	2689.73	2.57E-95–2.81E + 101	0.945	0.893
CD14 CD206 > 1.8	22	0.61	0.07–5.4	0.659	0.786
CD14 TLR9 > 1.3	12	1.9e3	7.0e-42–5.2e47	0.885	1.000
CD14 fractalkine > 0.7	12	1.8e5	0.00–Inf	0.988	1.000
C14hi CD16lo > 5	35	13.93	2.86–67.91	0.001*	0.780
CD14hi CD16lo CD64 > 4.6	35	4.62	1.50–14.21	0.008*	0.778
CD14hi CD16lo CCR2 > 4.8	35	13.93	2.86–67.91	0.001*	0.780
CD14hi CD16hi > 0.5	33	2.03	0.67–6.13	0.209	0.730
CD14hi CD16hi CD64 > 0.5	33	2.79	1.07–7.33	0.037*	0.746
CD14hi CD16hi CCR2 > 0.2	33	1.22	0.47–3.17	0.685	0.712
**ADJUSTED CD4 THRESHOLDS**					
CD4 > 10	35	3.63	1.13–11.69	0.031*	0.795
CD4 CD28hi > 8.7	35	4.42	1.34–14.57	0.015*	0.762
CD4 CD28lo > 4.7	35	0.59	0.18–1.92	0.378	0.732
CD4 CD25 > 0.74	35	3.13	1.17–8.39	0.023*	0.756
CD4 MHC class II > 0.11	35	0.64	0.17–2.41	0.511	0.722
CD4 CD154 > 0.03	35	2.08	0.26–16.4	0.487	0.730
**ADJUSTED CD8 THRESHOLDS**					
CD8 > 2	35	3.35	1.09–10.28	0.035*	0.770
CD8 CD28hi > 1.8	35	4.39	1.12–17.14	0.033*	0.760
CD8 CD28lo > 1.9	35	2.17	0.81–5.82	0.125	0.742
**ADJUSTED CCR4 AND CXCR3 THRESHOLDS**					
CCR4 > 6	26	0.38	0.13–1.12	0.079	0.765
CCR4 CD4 > 7.6	25	0.53	0.09–3.18	0.488	0.726
CCR4 CD8 > 2.7	26	0.22	0.02–2.00	0.178	0.768
CXCR3 > 7.5	26	1.57	0.49–5.04	0.452	0.747
CXCR3 CD4 > 10.3	26	5.28	0.68–40.79	0.111	0.713
CXCR3 CD8 > 1.3	26	16.48	2.78–97.56	0.002*	0.826

### Longitudinal analyses of significant biomarkers are highly variable

**Figure 4:**
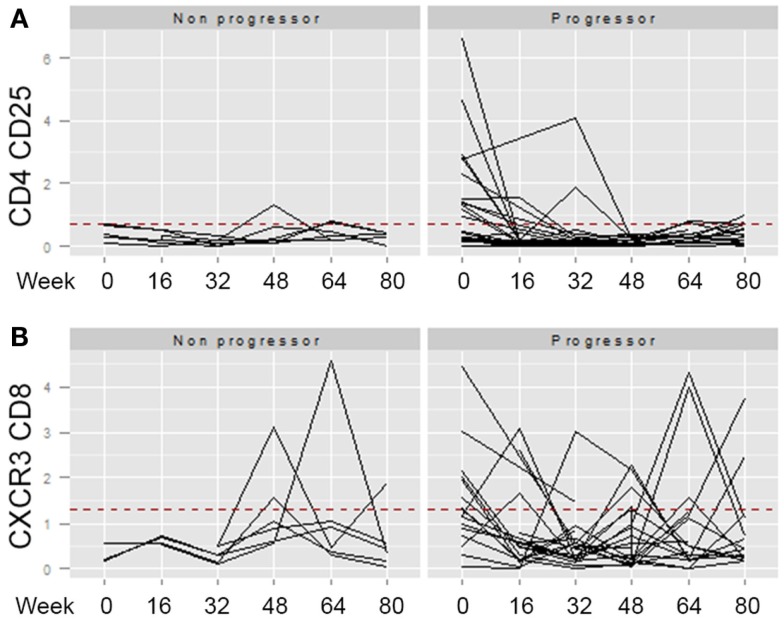
**Longitudinal analyses of T cell phenotypes are highly variable**. The percentage of CD4 CD25 **(A)** or CD8 CXCR3 **(B)** expressing T cells identified at longitudinal time points (weeks 0, 16, 32, 46, 64, and 80) were graphed. The dotted red line indicates the threshold above which this cell population was found to predict the patient reaching the combined endpoint in Table 5. For both of these markers, univariate and multivariate analyses of the percentages at baseline significantly separated non-progressors from progressors. However, elevation of the marker at baseline, did not predict stable elevation of the marker over time.

**Figure 5:**
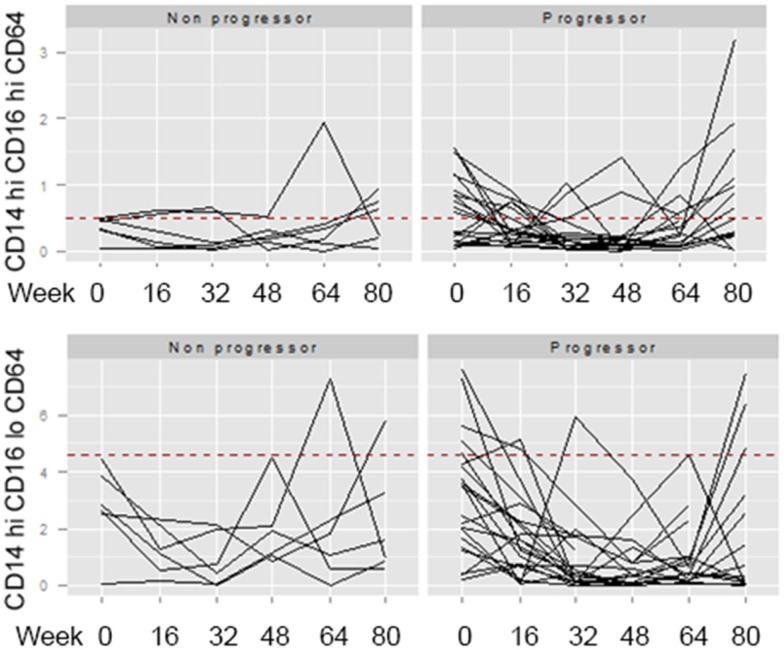
**Longitudinal analyses of monocyte phenotypes are highly variable**. The percentage of CD14hi CD16hi CD64 (top) or CD14hi CD16lo CD64 (bottom) expressing monocytes identified at longitudinal time points (weeks 0, 16, 32, 46, 64, and 80) were graphed. The dotted red line indicates the threshold above which this cell population was found to predict the patient reaching the combined endpoint in Table 5.

Across the univariate, multivariate, and threshold analyses, percentages of both CD4 CD25 and CD8 CXCR3 expressing T cells measured at baseline predicted IPF disease progression and worse survival (Figures 3C,D). To determine whether elevations in these cellular phenotypes were stable across the 80-week observation period, longitudinal spaghetti plots were generated. Figure 4 demonstrates that percentages of these cellular phenotypes were variable with time. As expected, levels detected in non-progressive patients were generally below the threshold limit. However, in the progressive patients, elevations in the percentages of these cell types were not maintained over time. To determine whether the monocyte phenotypes associated with disease progression in our threshold analyses also showed a similar variation, we performed a similar longitudinal analysis for the CD14 hi CD16hi CD64 and the CD14hi CD16lo CD64 expressing monocytes. Again, as expected, the non-progressive patients generally had percentages that were below the threshold limit at all time points (Figure 5). However, like the T cell analyses, the progressive IPF patients demonstrated highly variable percentages of the cell types over time.

## Discussion

This study was undertaken to determine whether activated leukocyte phenotypes at baseline could predict which IPF patients were at risk of disease progression. We chose to analyze T cell markers based on three previous studies that had indicated activated T cells characterize IPF patients (7–9). In two of these studies, the activated T cell phenotypes were associated with worse outcomes (7, 9). Furthermore, the study by Gilani et al. suggested that IPF patients were characterized by low levels of CD28 on CD4 T cells, results that the authors interpreted to indicate persistent antigen activation. Based on this, we were surprised that in our multivariate Cox-models in Table 4, it was the CD4 CD28hi population of T cells that correlated with progression in our study. While loss of CD28 can be a marker of clonal exhaustion, upregulation of CD25, MHC class II, and CD154 are also indications of T cell activation. As we did note that elevated percentages of CD4 CD25 (in all analyses) correlated with disease progression, and CD4 MHC class II expressing CD4 cells approached significance in multivariate analyses, we believe that the progressive IPF patients are characterized by activated T cells measured at baseline. Figure 3C highlights the rapid probability of decline in PF survival noted in patients with levels of CD4 CD25 expressing T cells over the 0.74% threshold in peripheral blood. CD25 can also be a marker of regulatory T cells as well as activated T cells. Unfortunately, our analyses did not include assessment of FoxP3, which would have more definitively differentiated these two possibilities. It is not clear why our results differ from those of Gilani et al., but one possibility is that our patients were not as advanced in their disease process. In the Gilani et al. study, the IPF patients were older and had worse baseline lung function than in our cohort (7). Thus, it is possible that our cohort of IPF patients have not yet achieved the levels of T cell clonal exhaustion noted in their study. Another possibility is that expression levels in our cohort may have been affected by the overnight shipment of samples from multiple centers rather than being carried out on the day of blood draw. Finally, it is possible that the inherent heterogeneity of the measurement of these markers when analyzed over time (as demonstrated in Figure 4 for the CD4 CD25 fraction of cells) may explain such discrepancies.

Further support for our interpretation that progressive IPF patients are characterized by activated T cells comes from the knowledge that the CXCR3 chemokine receptor is highly expressed on activated T cells, and in particular T cells, which can migrate into inflamed tissues such as rheumatoid synovium (28). However, it is interesting to note that our findings of increased percentages of CXCR3+CD8+ cells predicting disease progression in IPF patients are at odds with a previous murine study and two immunohistochemical studies of lung lymphocytes (15, 29, 30). In the study by Jiang et al., CXCR3-deficient mice were shown to have worse experimentally induced lung fibrosis (29). This was associated with a failure to generate IFNγ in response to bleomycin-injury. Furthermore, a previous study suggested that CCR4 expression correlates with Th2 activation while CXCR3 expression correlates with Th1 cell activation and suggested that IPF patients are characterized by relatively equal ratios of CXCR3 and CCR4 expressing T cells whereas NSIP patients who have better prognoses are characterized by elevated expression levels of CXCR3. (15). In our study, IPF progressors had higher ratios of CXCR3 to CCR4 expression, suggesting Th1 activation. Furthermore, patients with levels of CXCR3 expressing CD8 T cells >1.3% of the peripheral blood had rapid PF survival decline (Figure 3D). The differences in our study and the previous study could be explained by methodology. The previous study stained sections of lung tissue while our results examined peripheral blood. It is likely that the chemokine profiles of tissue-recruited lymphocytes could be quite different. In support of this, Pignatti et al. confirmed that bronchoalveolar lavage cells from IPF patients had lower levels of CXCR3 than CCR4 expressing T cells (30); however, these same differences were not noted in peripheral blood. This highlights the tissue-specific differences in chemokine receptors which seem to mark lung vs. circulating T cells. While it is not clear why the association with circulating CXCR3 CD8 cells and IPF disease outcome is so great in our cohort, we interpret the T cell results overall to suggest that there is a strong inflammatory response present at baseline in the patients who progress. The fact that the activated T cell phenotypes do not persist longitudinally (Figure 4) may suggest that we “caught” some IPF patients during an episodic inflammatory event. Given the 80 weeks of follow-up, we had the greatest power to detect progression in patients who were experiencing this inflammatory event at week 0. It is possible that elevations in any of these markers at later time points in patients we classified as non-progressors may actually be predictive of their eventual decline, but because our analyses stopped after 80 weeks, we had insufficient clinical data to make such a determination.

Another interesting finding in our study was the observation that increases in CD14hi CD16hi expressing cells at baseline correlated with disease progression in both univariate and multivariate analyses. As expected, this population largely expresses not only CD64 but also CCR2. The murine equivalent of this subset of monocytes has previously been suggested to promote myofibroblast differentiation (22). While the association with this subset of monocytes and disease progression was consistent throughout all of our analyses, it should be noted that this cohort of cells represents a relatively small fraction of the total peripheral blood pool. Patients with CD14hi, CD16hi, CD64+ cells above 0.5% of peripheral blood at baseline had worse PF survival outcomes (Figure 3B), but these percentages varied considerably over time (Figure 5). Certainly, the paracrine factors secreted by such cells could have profound impact on resident lung mesenchymal cells even if present in small numbers.

It is interesting that in the univariate, multivariate, and threshold analyses, percentages of CD14+CD206+ cells did not correlate with disease progression. These data were surprising to us as alternatively activated macrophages (defined by expression of the CD206 mannose receptor or CD163 scavenger receptor) are believed to be associated with IPF (20, 31). In mouse models, depletion of the Ly6Chi population was shown to reduce the degree of alternatively activated macrophages found in lung tissue and their adoptive transfer could promote fibrosis even though the Ly6Chi monocytes were not found in the lung tissue. Extrapolating to the human circumstance, this would suggest that the CD14hi CD16lo population would correlate with development of the CD206+ alternatively activated macrophages. While the univariate analyses did show an increased hazard ratio (HR = 1.57) for patients with increases in the CD14hi, CD16lo, CCR2+ cells, the significance was not maintained in the multivariate analyses and this may explain our inability to demonstrate an association with alternatively activated monocytes in our relatively small cohort of IPF patients.

In summary, our results are consistent with studies suggesting that the presence of activated lymphocytes in the blood of IPF patients. Patients who had elevated levels of CD4 CD25, CD8 CXCR3, and various subsets of both CD14hi CD16hi and CD14hi CD16lo monocytes all demonstrated disease progression. The fact that these biomarkers were not stably elevated over time, suggests that IPF patients experience episodic inflammatory events (perhaps driven by occult infections), which ultimately mark them for disease progression. Our documentation of biomarker heterogeneity over time complicates the interpretation of these markers for determining patient outcomes. Certainly, patients who display such elevations may be appropriate for enrollment in therapeutic trials, but clearly the absence of such markers at baseline does not guarantee stability. Thus, it may be more appropriate to serially monitor IPF patients for a panel of inflammatory and activated leukocyte phenotypes in order to predict the likelihood that they will progress following such an episode. In this regard, identifying appropriate thresholds for triggering such declines will be crucial, and will take a much larger study. Interestingly, a recent study looking at longitudinal variability in circulating fibrocytes in Hermansky–Pudlak patients with interstitial lung disease also indicated episodic elevations over time, but found threshold levels for the highest measurement ever that predicted mortality (32). A caveat to the interpretation of our study is that we did not protocolize the collection of information regarding infection, on-going smoking status, or current treatments thus, some of our observed variability may be related to unknown and non-standardized therapeutics. We cannot determine whether any of these factors influenced our measurements over time. It is also interesting to speculate that IPF patients may decline because they are not able to sufficiently activate their innate and adaptive leukocytes. While immunosuppressive therapy has shown benefit in some interstitial lung diseases (4, 5), the results are somewhat controversial because of the recent study, which found that immunosuppressive agents do not benefit patients with IPF (6). While there may be differences in the cohorts used in all these studies, we believe, based on our current results that it is interesting to speculate that immune stimulation may have some benefit.

## Author Contributions

Collected samples: Fernando J. Martinez, MeiLan K. Han, and Kevin R. Flaherty. Processed samples, performed flow cytometry: Bethany B. Moore and Chris Fry. Analyzed data and interpreted results: Bethany B. Moore, Yueren Zhou, Susan Murray, Fernando J. Martinez, and Kevin R. Flaherty. Prepared manuscript: Bethany B. Moore, Yueren Zhou, Susan Murray, and Kevin R. Flaherty. Edited manuscript: Bethany B. Moore, MeiLan K. Han, Yueren Zhou, Susan Murray, Fernando J. Martinez, and MeiLan K. Han.

## Conflict of Interest Statement

The authors declare that the research was conducted in the absence of any commercial or financial relationships that could be construed as a potential conflict of interest.
